# Effect of Sleep Bruxism Intensity on Blood Pressure in Normotensives

**DOI:** 10.3390/jcm10061304

**Published:** 2021-03-22

**Authors:** Monika Michalek-Zrabkowska, Mieszko Wieckiewicz, Pawel Gac, Joanna Smardz, Rafal Poreba, Anna Wojakowska, Katarzyna Goslawska, Grzegorz Mazur, Helena Martynowicz

**Affiliations:** 1 Department of Internal Medicine, Occupational Diseases, Hypertension and Clinical Oncology, Wroclaw Medical University, 213 Borowska St., 50-556 Wroclaw, Poland; monika.michalek@student.umed.wroc.pl (M.M.-Z.); rafal.poreba@umed.wroc.pl (R.P.); anna.wojakowska@umed.wroc.pl (A.W.); k.goslawska@hotmail.com (K.G.); grzegorz.mazur@umed.wroc.pl (G.M.); 2 Department of Experimental Dentistry, Wroclaw Medical University, 26 Krakowska St., 50-425 Wroclaw, Poland; mieszko.wieckiewicz@umed.wroc.pl (M.W.); joanna.smardz@umed.wroc.pl (J.S.); 3 Department of Hygiene, Wroclaw Medical University, 7 Mikulicza-Radeckiego St., 50-345 Wroclaw, Poland; pawel.gac@umed.wroc.pl

**Keywords:** sleep bruxism, ambulatory blood pressure monitoring, polysomnography

## Abstract

The present research aimed to investigate the influence of sleep bruxism (SB) intensity on blood pressure parameters in normotensive subjects by using an ambulatory blood pressure device. The study group included 65 normotensive individuals suspected of having SB. All participants underwent one-night video-polysomnography, and ambulatory blood pressure monitoring was performed the next day; 86.15% of them were diagnosed with SB. Statistical analysis included correlation and regression analysis. The obtained results showed that systolic blood pressure variability during sleep significantly increased in individuals with BEI > 4 (bruxism episodes index; episodes/hour) compared to those with BEI ≤ 4 (8.81 ± 3.36 versus 10.57 ± 3.39, *p* = 0.05). Multivariable regression analysis showed that systolic blood pressure variability at nighttime was also associated with the following independent risk factors: higher apnea-to-bruxism index, male gender, BEI > 4 episodes/hour, body mass index (BMI) ≥ 25 kg/m^2^, higher arousal index, and shorter total sleep time. In summary, sleep bruxism intensity was associated with increased systolic blood pressure variability during sleep. Coincidental apnea, male gender, severe sleep bruxism (SB intensity with BEI > 4/hour), excess weight and obesity, higher arousal index, and shorter sleep time seem to be the main determinants that influence blood pressure in normotensive sleep bruxers.

## 1. Introduction

Nocturnal drop in blood pressure (BP) is a physiological component of circadian rhythm. [1] The dipping of BP at nighttime is an effect of sympathetic tone decrease during sleep. Absent or diminished decrease in BP during sleep is associated with increased cardiovascular mortality and morbidity [2,3]. Arterial hypertension (HTA) has been reported to be linked with a large number of sleep disturbances: short sleep duration [4], insomnia [5], restless leg syndrome [6], and obstructive sleep apnea (OSA) [7].

Sleep bruxism (SB) is a common sleep-related disorder. The etiology of SB is multifactorial and is still insufficiently explored. Several theories on sleep bruxism etiology and pathophysiology have been proposed; some of these theories focus on a genetic basis and neurotransmission [8], while others focus on psychological risk factors or sympathetic hyperreactivity. The prevalence of SB in adults is estimated between 3% and 8% [9,10]. Clinical manifestations of SB include tooth grinding and/or jaw clenching, abnormal tooth wear, and jaw muscle pain due to the fact of jaw muscle activity (rhythmic masticatory muscle activity (RMMA)) during sleep and are classified in the 3rd edition of the International Classification of Sleep Disorders [11].

As shown in the literature, SB commonly coexists with obstructive sleep apnea (OSA) [12]. A recent systematic review by Van Ryswyk et al. [12] examined a large number of epidemiological studies on the relationship between sleep-related breathing disorders and hypertension. As explored in a cross-sectional, multicenter Sleep Heart Health Study (SHHS), the odds ratio increased and was estimated to be 1.37 (95% confidence interval (CI), 1.03–1.83) for hypertension for subjects with severe OSA compared to that in individuals without OSA [13]. The relationship between OSA and HTA was also confirmed by the Wisconsin Study [14] and the Zaragoza Sleep Cohort Study [15]. The pathomechanism linking increased mortality in apneic patients with HTA refers to BP variability (BPV), with a gradual rise in BP at the beginning of an apneic/hypopneic event and then abrupt fall in BP after the respiratory event [16]. Recurrent episodes of hypoxic events cause increased cardiovascular morbidity and mortality due to the oxidative stress [17].

Previous studies have emphasized the role of increased sympathetic activity preceding the SB event [18]. A repetitive surge in sympathetic activity expressed as increased heart rate followed by an increase in electroencephalography (EEG) frequency (microarousal) precedes RMMA [19]. Correspondingly, an autonomic sympathetic activation is a key component of pathophysiological repercussions of HTA, for example, vascular, cardiac, renal, metabolic, and immune-related abnormalities [20].

As previously reported in the literature, HTA is correlated with sleep bruxism. A seminal contribution has been made by Nashed et al. [21], who revealed that BP fluctuations during sleep were associated with RMMA. In another study by Martynowicz et al. [22], bruxism intensity was found to be increased in hypertensives. It is worth noting that the design of this study was focused on the assessment of intensity (frequency) and prevalence of SB in patients with HTA compared to that in non-hypertensive subjects.

To date, only a few studies have investigated the association of SB with hypertension. To fill this gap in the literature, the current descriptive study aimed to assess the relationship between ambulatory blood pressure measurements and SB intensity in individuals without HTA.

## 2. Materials and Methods

Sixty-five subjects suspected of having SB were admitted to the Department of Internal Medicine, Occupational Diseases, Hypertension, and Clinical Oncology at the Wroclaw Medical University in Poland. Before admission, all participants underwent complex dental examination at the Clinic of Prosthetic Dentistry at Wroclaw Medical University. The dental examination involved physical examination (focusing on clinical indicators of bruxism [23]) and a medical interview. Individuals who met the criteria for the presence of probable SB according to an international consensus on the assessment of bruxism were included in the study group [24].

The inclusion criteria for the study were as follows: age above 18 years, diagnosis of probable SB based on dental examination, and willingness to participate in the study. The exclusion criteria were diagnosis of HTA or use of anti-hypertensive medications; use of medicines affecting the nervous and muscular systems; cognitive disabilities and inability to provide informed consent; inability to undergo polysomnography (PSG) or measurement with an ambulatory blood pressure monitoring (ABPM) device; severe physical and mental conditions; presence of diabetes, coronary artery disease, active malignancy, or inflammation.

All participants signed a consent form and voluntarily underwent examination. The study was approved by the local Ethics Committee (no. KB-195/2017) and was conducted by adopting the principles of the Declaration of Helsinki. This clinical trial was also registered in Clinical Trials Database (www.ClinicalTrials.gov, accessed on: 20 March 2021): identifier NCT03083405, WMU1/2017.

Polysomnographic examinations were conducted in the sleep laboratory at the Department of Internal Medicine, Occupational Diseases, Hypertension, and Clinical Oncology at the Wroclaw Medical University in Poland. The PSG data were collected using a Nox-A1 (Nox Medical, Reykjavík, Iceland) device for one-night video-PSG. Recordings included sleep, bruxism, and respiratory data as follows: sleep latency, total sleep time, and sleep efficiency (%); the ratio of N1 (sleep stage 1), N2 (sleep stage 2), N3 (sleep stage 3), and the stage of REM (rapid eye-movement sleep); electromyographic (EMG) recordings of bilateral masseter muscle activity during sleep; video and audio recordings; respiratory events recorded by a nasal pressure transducer; and arterial oxygen saturation (SpO_2_) measured by a finger pulse oximeter.

After conducting an automatic analysis, 30 s epochs of polysomnograms were evaluated by a certified polysomnographist according to the gold standard criteria of the American Academy of Sleep Medicine (AASM) task force [25]. Respiratory events were scored as follows: the absence of airflow for ≥10 s was scored as an apneic event, while a reduction in the amplitude of breathing by ≥30% for ≥10 s with a ≥3% decline in blood oxygen saturation or arousal was scored as hypopnea. Sleep bruxism was assessed audio-visually from EMG measurements supplemented by audio and video recordings. The increase in EMG amplitude that was at least twice that of the background EMG was considered as a bruxism episode. Electromyographic bursts within 3 s were considered to be the part of the same episode. The bruxism episode index (BEI) was determined as the number of bruxism events per hour of sleep, and the SB intensity was classified as insignificant (BEI < 2), mild or moderate (BEI = 2–4), or severe (BEI > 4) [23]. The apnea-to-bruxism index refers to a bruxism event secondary to a respiratory event. Temporal association between SB and respiratory events were demonstrated and evaluated previously, suggesting that SB events were secondary to apneic–hypopneic events [26]. As there are no reference values, the authors assumed an apnea-to-bruxism index of <1 as normal, concluding that an apnea-to-bruxism index greater than 1 may suggest other etiology of bruxism events, e.g., primary bruxism or against-hypoxia protective features of SB.

The study group consisted of normotensive individuals. Normotension was defined as the lack of use of any antihypertensive medications in medical history and was based on the office BP measurements. According to European guidelines for the management of HTA, individuals with BP values of at least 140/90 mmHg were considered as normotensive [27]. All patients included in the study had an office BP measurement in the normal range at the day of admission to internal ward. On the first night, individuals underwent full-night video-polysomnography. Blood pressure measurements were conducted the next day. After conducting PSG, in the morning, patients received ABPM for 24 h home use as a mandatory requirement for the diagnosis of hypertension. The ABPM devices were attached by a qualified technician, and the measurement was performed according to recommendations of European Society of Hypertension [28]. First, the technician measured the circumference of the upper arm and selected the appropriate cuff. Next, all participants were instructed about the correct position during BP measurement, and the technicians read through the instruction paper together with the patient before 24 h measurements and explained the possible hazards in detail on the basis of the warnings included in the instructions. The ABPMs were conducted with validated Spacelabs OnTrak Ambulatory Blood Pressure Monitor appliances. The measurement intervals estimated were 15 and 20 min during daytime and nighttime periods, respectively. Daytime was recorded as the period between the time of attachment of the device by the patient and the time when the patient went to bed. Nighttime was registered as the period between the time when the patient went to bed and the time when the patient woke up in the morning. Subsequently, the devices were collected from patients on the following day. The ABPM measurements were scored according to the cut off values in the ESH/ESC (the European Society of Hypertension and the European Society of Cardiology) guidelines for 2018 [27] and extrapolated with findings from PSG.

Statistical analyses were performed using Dell Statistica 13.1 statistical package (Dell Inc., Round Rock, TX, USA). The distribution of variables was assessed with the W-Shapiro–Wilk test. For independent quantitative variables with normal distribution, the *t*-test was used for further statistical analysis. For variables with non-normal distribution, the Mann–Whitney U test was used. Correlation and regression analyses were performed to determine the relationship between the studied variables. The model parameters obtained in the regression analysis were estimated using the least squares method. The results at the level of *p* < 0.05 were considered to be statistically significant.

## 3. Results

The study group consisted of 65 normotensive individuals; the mean age of the participants was 33.96 ± 11.22 years, and 20 (30.77%) were men and 45 (69.23%) were women. The average body mass estimated was 65.12 ± 13.22 kg, the mean height was 1.70 ± 0.08 m, and the average body mass index (BMI) (calculated as weight in kilogram divided by square of height in meters) was 22.48 ± 3.87 kg/m^2^. According to BMI, three subjects were identified to be underweight (BMI < 18.5 kg/m^2^), 33 had normal weight (BMI = 18.5–25 kg/m^2^), nine were overweight (BMI = 25–30 kg/m^2^), and three were obese (BMI > 30 kg/m^2^).

After one-night PSG, 56 (86.15%) participants met the criteria for SB, of which 25 were diagnosed with mild/moderate SB (BEI = 2–4 events/h) and 31 had severe SB (BEI > 4 events/h). On the basis of the apnea–hypopnea index (AHI), 14 (21.54%) participants were diagnosed to have OSA, including eight (12.31%) with mild OSA (AHI = 5–15 events/h), three (4.62%) with moderate OSA (AHI = 15–30 events/h), and three (4.2%) with severe OSA (AHI > 30 events/h).

None of the patients in the study group met the criteria for accompanying diseases such as hypertension, diabetes mellitus, or coronary artery disease. The polysomnographic and ABPM parameters in the studied group are presented in Table 1 and Table 2.

The difference in the ambulatory BP data among participants with BEI < 2 and those with BEI > 2 was nonsignificant, but the group of non-bruxers was relatively small (*n* = 9). However, significant differences in ABPM findings were observed between severe bruxers and the remaining study group (Table 3).

Differences in the ambulatory BP data between individuals with an apnea-to-bruxism index < 1 and those with an apnea-to-bruxism index ≥ 1 was statistically significant (Table 4).

Regression analysis was performed to determine the factors independently associated with mean arterial pressure (MAP) (average from the 24 h mean of APBM and daytime measurements) and systolic blood pressure (SBP) variability during sleep period. In the regression model, we used all potentially independent variables: apnea-to-bruxism index, male gender, BMI, age, BEI > 4, BMI ≥ 25, arousal index, and total sleep time (TST).

The regression model showed that a higher apnea-to-bruxism index and higher BMI were independently associated with the increased average of 24 h mean of MAP (Table 5).

This finding confirmed that a higher apnea-to-bruxism index and higher BMI were independent predictors of the increased average MAP during daytime in the studied group (Table 6).

The regression analysis also indicated that a higher apnea-to-bruxism index, male gender, BEI > 4, BMI ≥ 25 kg/m^2^, higher arousal index, and shorter TST were the independent risk factors for increased SBP variability during sleep period (Table 7 and Table 8).

## 4. Discussion

The results of the current study showed an association between SB intensity and blood pressure fluctuations. The results demonstrate that SB affects BP. The SBP variability at nighttime significantly increased in severe sleep bruxers as compared to those in the remaining study group. Blood pressure variability (BPV) reflects dynamic changes in BP caused by physical, emotional, and environmental factors. The clinical importance of BPV is broadly discussed as follows. BPV is considered a risk factor for cardiovascular events [29,30] and is associated with organ complications and cardiovascular mortality [29,31] independent from mean 24 h BP values. Therefore, cardiovascular reactivity and BP fluctuations promote an individual’s risk of developing hypertension and can be considered to be equivalent to the traditional determinants of hypertension. Thus, the implications of the current findings involve serious risk of hypertension development in severe sleep bruxers without the actual diagnosis of hypertension. Moreover, regression analysis demonstrated that SB intensity was associated with increased SBP variability independently from anthropometric and polysomnographic parameters such as age, gender, BMI, and apnea events. Importantly, the current findings show a novel association between SB intensity and SBP variability independent from OSA. Lower minimal values of SBP at nighttime in severe sleep bruxers as compared to that in the remaining study group may be considered due to the sympathetic tone balance as argued previously [32,33].

This result highlights that little is known about the implications of SB for general health. Metabolic and hormonal disturbances related to the increased level of inflammatory and stress markers were found in young sleep bruxers in a prior study by our research team [34]. Several existing studies in the broader literature have examined clinical implications of SB on oral health and sleep-related disorders [9,23,35]. Seminal contributions to more comprehensive perspectives have been made by Huynh and Lavigne [18,19,36] who investigated the role of autonomic cardiac activation in SB events. Finally, the authors demonstrated the co-occurrence of RMMA events, arousal, and respiratory events in patients with SB [37,38].

The literature on the relationship between SB and hypertension confirmed our results. For instance, Nashed et al. [21] demonstrated that RMMA is associated with BP fluctuations during sleep. The methods were based on nocturnal beat-to-beat BP measurements. Although the study was well conducted and designed, the study group consisted of only 10 participants, whereas the control group had nine individuals. In a previous study by our research team, Martynowicz et al. [22] demonstrated that hypertension was an independent risk factor for SB. The lack of objective diagnosis of hypertension was the most important limitation of this previous study; hence, we designed the current study to diagnose hypertension and assess cardiovascular risk in bruxing, normotensive healthy patients by using ABPM.

It is also important to highlight the genetic basis of SB. The influence of neurotransmitters involved in sleep regulation, such as acetylcholine, noradrenaline, dopamine, and serotonin, on the genesis of SB has been insufficiently explored [39,40]. Wieckiewicz et al. [8] suggested that the genetic basis of primary bruxism is associated with the rs686 G variant of the dopamine receptor DRD1 encoding gene and the rs2770304 and rs6313 polymorphisms of the serotonin receptor HRT2A encoding gene. Their study also revealed a possible genetic association between SB and OSA, wherein a statistically significant correlation was observed within a group of HTR2A rs2770304 polymorphism TT homozygous cases. The serotonin receptor HRT2A has been linked previously with stress-induced response and anxiety-level mediation [41]. Serotoninergic mechanisms seem to play a crucial role in SB pathogenesis [8]. Therefore, the potential mechanism that causes decrease and variability in SBP in severe sleep bruxers could reflect the regulating effect of serotonin on the autonomic system activity [42]. Serotonin is also related to vasoconstriction and vasodilatation functions [43]. The results of the current study support this fact by showing that severe sleep bruxers had lower MAP in daytime. This finding is consistent with the assumption that nocturnal BP fluctuations cause dysregulations of the cardiovascular system.

A further relevant result is the correlation between BP measurements and SB events related to apnea. The study group included relatively young, normotensive subjects (mean age 33.96 ± 11.22 years) without severe comorbidities; therefore, the prevalence of OSA in this study group was considered to be a surprising finding. It is difficult to explain this result within the context of the normal weight of the study group (average BMI: 22.48 ± 3.87 kg/m^2^). Obesity is the major risk factor for OSA [44]. The remaining risk factors for OSA are well documented and include a family history of OSA, retrognathia, resistant hypertension, congestive heart failure, atrial fibrillation, stroke, and type 2 diabetes [45]. None of the participants from the study group met these criteria (risk factors). Thus, the high prevalence of OSA (21.54%) in the study group without classical risk factors for OSA may suggest a secondary SB related to apneic events. This relationship was previously observed by Hosoya et al., Martynowicz et al., and Tan et al. All these authors have highlighted the protective features of SB against hypoxic event [46,47,48]. Moreover, the literature on SB has established sympathetic activity as the underlying reason for its consequences such as heart rate variability, hypertension, OSA, and hormonal and metabolic disturbances. A significant difference in the ABPM data between participants with an apnea-to-bruxism index < 1 and those with an apnea-to-bruxism index ≥ 1 supports this hypothesis. In the group with higher number of SB events related to apnea, increased BP values were found. Bruxism related to apnea events influenced both daytime and nighttime measurements of the increased BP values. In contrast, heart rate significantly decreased in the study group with an apnea-to-bruxism index ≥ 1. A recent study of our research team provided some information related to the background of this issue and reported heart rate variability as an expression of sympathetic tone in SB and its consequences. [33] A significant decrease in heart rate was considered as a consequence of autonomic homeostasis in response to increased cardiac activity. Xu et al. demonstrated that in a study group with severe OSA, nocturnal and awake BP values were associated with the duration of hypoxia during sleep and BP fluctuations. In contrast to our present study design, BP was assessed in this study by measuring pulse transmit time (PTT) [49].

The broad implication of the present research is that SB in young patients with increased sympathetic tone favors the development of BP fluctuations [21] and subsequently hypertension. Sympathetic activity is modulated by an increased level of stress and anxiety, which are the major determinants of SB. Subsequently, contributory risk factors, such as age, gender, and weight gain, influence and determine OSA and hypertension progression, which are associated with SB. [47] The very basis of this positive feedback loop is sympathetic activity. The cause-and-effect relationship between increased sympathetic activity and blood pressure fluctuations in sleep bruxers suggest association between SB and increased risk of cardiovascular complications. Despite previous and current study are preliminary, authors suggest assessing cardiovascular and respiratory implications of SB in bruxing patients. Further studies should aim to provide more information about this health prevention concern.

The present study had some limitations. First, the results are specific to young, healthy, normotensive adult subjects, and we did not diagnose elderly patients or children. It could also be argued whether BP fluctuations in sleep bruxers could affect co-existing cardiovascular conditions. Further studies on comparison between SB patients with cardiovascular diseases and controls are needed to determine whether SB affects BP alterations and worsens CVD. Future studies should aim to replicate the results in vulnerable individuals. Second, there was a lack of parity in the study group, and women constituted 69.23% of the study participants. This apparent limitation could influence our findings, as has been revealed in a recent study by Smardz et al. [50] where age and gender were found to influence SB. Last but not least, the participants underwent one-night PSG without an adaptation due to the presence of technical issues.

To summarize, the results of our present study shed new light on SB comorbidity, especially BP fluctuations and warrants a change in the clinical approach to patients with SB.

## 5. Conclusions

1. SB intensity is associated with increased SBP variability at nighttime (sleep period);
2. Coexisting apnea is considered to be the main determinant that influences BP in normotensive sleep bruxers;
3. A future approach should consider the potential risk of hypertension or abnormalities in BP regulation and other cardiovascular risk factors in sleep bruxers more carefully as potentially significant implications for health.

## Figures and Tables

**Table 1:** Polysomnographic characteristics of the study group.

Parameter	Mean	SD
AHI (*n*/h)	5.07	8.62
ODI (*n*/h)	4.76	7.48
Snore (% of TST)	7.35	14.74
TST (min)	428.00	65.50
SL (min)	22.76	21.91
REML (min)	100.03	62.31
WASO (min)	31.12	31.19
SE (%)	86.30	10.60
N1 (% of TST)	4.48	3.75
N2 (% of TST)	49.92	7.59
N3 (% of TST)	22.65	7.02
REM (% of TST)	22.95	5.26
Arousals (n/h)	5.04	3.92
Mean SpO_2_ (%)	94.65	1.80
Min SpO_2_ (%)	88.62	6.16
SpO_2_ < 90% (%)	1.48	4.74
Mean desaturation drop (%)	3.23	0.60
Mean heart rate (beats/min)	60.67	6.84
Max heart rate (beats/min)	95.97	11.79
Min heart rate (beats/min)	48.52	5.98
BEI (*n*/h)	5.06	3.54
Phasic (*n*/h)	3.86	7.27
Tonic (*n*/h)	2.34	2.29
Mixed (*n*/h)	1.30	1.59
Apnea-to-bruxism index	1.09	1.99

AHI, apnea–hypopnea index; ODI, oxygen desaturation index; TST, total sleep time (min); SL, sleep latency; REML, REM latency; WASO, wake after sleep onset; SE, sleep efficiency; N1, sleep stage 1; N2, sleep stage 2; N3, sleep stage 3; REM, rapid eye-movement sleep stage; mean SpO_2_, mean oxygen saturation (%); SpO_2_ < 90%, time with oxygen saturation < 90% (% of TST); BEI, bruxism episode index.

**Table 2:** Ambulatory BP characteristics of the study group (values are shown as mean ± SD).

Variable		24 h Mean	Daytime	Nighttime
SBP	Average (mmHg)	110.56 ± 7.91	114.03 ± 8.78	101.68 ± 7.94
	Variability (mmHg)	12.61 ± 2.99	11.09 ± 2.68	9.66 ± 3.46
	Minimum (mmHg)	84.75 ± 7.43	90.59 ± 9.46	85.55 ± 8.05
	Maximum (mmHg)	141.88 ± 14.10	141.67 ± 14.07	121.75 ± 12.92
	Decline (%)	10.67 ± 6.06		
DBP	Average (mmHg)	68.83 ± 6.08	72.31 ± 7.12	60.60 ± 6.55
	Variability (mmHg)	11.19 ± 2.65	9.98 ± 2.89	8.48 ± 3.00
	Minimum (mmHg)	46.81 ± 6.58	51.73 ± 8.08	47.15 ± 6.42
	Maximum (mmHg)	99.64 ± 15.29	99.09 ± 14.91	78.25 ± 12.26
	Decline (%)	15.75 ± 7.24		
MAP	Average (mmHg)	83.16 ± 6.11	86.13 ± 6.82	75.37 ± 6.37
	Variability (mmHg)	10.79 ± 2.42	9.74 ±2.53	8.07 ± 2.99
	Minimum (mmHg)	61.56 ± 6.49	65.61 ± 7.47	62.17 ± 6.63
	Maximum (mmHg)	113.28 ± 14.07	112.39 ± 13.87	91.97 ± 12.33
	Decline (%)	12.49 ± 6.00		
PP	Average (mmHg)	41.77 ± 4.97	42.02 ± 5.21	41.03 ± 5.03
	Variability (mmHg)	7.24 ± 1.95	7.45 ± 2.07	5.88 ± 1.81
	Minimum (mmHg)	23.80 ± 3.23	24.47 ± 4.16	29.35 ± 5.13
	Maximum (mmHg)	61.86 ± 9.97	61.00 ± 10.01	53.62 ± 8.31
HR	Average (beats/min)	72.09 ± 6.60	75.31 ± 7.24	63.35 ± 6.35
	Variability (beats/min)	12.82 ± 3.27	12.42 ± 3.69	7.20 ± 3.46
	Minimum (beats/min)	52.88 ± 5.98	55.39 ± 7.30	54.07 ± 5.32
	Maximum (beats/min)	112.55 ± 20.49	111.77 ± 20.10	83.62 ± 15.87

SBP, systolic blood pressure; DBP, diastolic blood pressure; MAP, mean arterial pressure; PP, pulse pressure; HR, heart rate.

**Table 3:** Ambulatory BP characteristics of patients with and without severe bruxism (BEI > 4 per hour).

	Variable		BEI ≤ 4	BEI > 4	*p*
SBP (mmHg)	24 h mean	Average	111.55 ± 8.42	109.52 ± 7.31	0.31
		Variability	12.59 ± 3.27	12.62 ± 2.71	0.97
		Minimum	86.39 ± 7.39	83.00 ± 7.19	0.07
		Maximum	143.97 ± 15.21	139.65 ± 12.67	0.22
		Decline (%)	10.87 ± 7.15	10.46 ± 4.75	0.80
	Daytime	Average	115.18 ± 9.54	112.81 ± 7.85	0.28
		Variability	10.86 ± 2.79	11.32 ± 2.58	0.50
		Minimum	92.21 ± 10.59	88.87 ± 8.04	0.16
		Maximum	143.97 ± 15.21	139.23 ± 12.52	0.18
	Nighttime	Average	102.45 ± 7.98	100.86 ± 7.95	0.44
		Variability	**8.81 ± 3.36**	**10.57 ± 3.39**	**0.05**
		Minimum	**88.13 ± 8.43**	**82.79 ± 6.72**	**0.01**
		Maximum	121.10 ± 13.04	122.45 ± 12.98	0.69
DBP (mmHg)	24 h mean	Average	68.97 ± 6.44	68.68 ± 5.78	0.85
		Variability	11.21 ± 2.76	11.17 ± 2.56	0.95
		Minimum	47.91 ± 6.78	45.65 ± 6.25	0.17
		Maximum	102.24 ± 15.98	96.87 ± 14.26	0.16
		Decline (%)	15.79 ± 8.88	15.71 ± 5.11	0.96
	Daytime	Average	72.09 ± 7.29	72.55 ± 7.04	0.80
		Variability	10.06 ± 3.01	9.91 ± 2.79	0.84
		Minimum	52.48 ± 8.19	50.94 ± 8.02	0.45
		Maximum	102.21 ± 16.00	95.77 ± 13.09	0.08
	Nighttime	Average	60.77 ± 7.13	60.41 ± 5.99	0.83
		Variability	7.99 ± 3.13	9.00 ± 2.82	0.20
		Minimum	48.52 ± 6.99	45.69 ± 5.50	0.09
		Maximum	77.71 ± 11.89	78.83 ± 12.83	0.73
MAP (mmHg)	24 h mean	Average	83.64 ± 6.35	82.65 ± 5.90	0.52
		Variability	10.86 ± 2.53	10.71 ± 2.34	0.80
		Minimum	62.33 ± 6.87	60.74 ± 6.07	0.33
		Maximum	116.09 ± 15.04	110.29 ± 12.50	0.10
		Decline (%)	12.62 ± 7.24	12.35 ± 4.45	0.87
	Daytime	Average	86.58 ± 7.37	85.65 ± 6.26	0.59
		Variability	9.83 ± 2.61	9.64 ± 2.49	0.76
		Minimum	66.24 ± 8.46	64.94 ± 6.32	0.49
		Maximum	**115.79 ± 15.04**	**108.77 ± 11.68**	**0.04**
	Nighttime	Average	75.81 ± 6.71	74.90 ± 6.07	0.58
		Variability	7.58 ± 2.92	8.59 ± 3.03	0.20
		Minimum	63.74 ± 7.16	60.48 ± 5.66	0.06
		Maximum	91.48 ± 12.28	92.48 ± 12.57	0.76
PP (mmHg)	24 h mean	Average	42.73 ± 5.37	40.74 ± 4.35	0.11
		Variability	7.36 ± 2.08	7.12 ± 1.82	0.63
		Minimum	24.00 ± 2.85	23.58 ± 3.62	0.61
		Maximum	62.97 ± 10.35	60.68 ± 9.57	0.36
	Daytime	Average	43.06 ± 5.65	40.90 ± 4.52	0.10
		Variability	7.61 ± 2.26	7.28 ± 1.87	0.53
		Minimum	24.73 ± 4.31	24.19±4.05	0.61
		Maximum	62.52 ± 10.64	59.39 ± 9.19	0.21
	Nighttime	Average	41.65 ± 5.24	40.38 ± 4.81	0.33
		Variability	5.58 ± 1.72	6.20 ± 1.89	0.19
		Minimum	30.48 ± 5.39	28.14 ± 4.63	0.08
		Maximum	52.87 ± 7.83	54.41 ± 8.87	0.48
HR (beats/min)	24 h mean	Average	72.24 ± 6.56	71.94 ± 6.76	0.85
		Variability	**13.64 ± 3.67**	**11.95 ± 2.57**	**0.04**
		Minimum	53.24 ± 6.17	52.48 ± 5.85	0.62
		Maximum	**117.82 ± 23.15**	**106.94 ± 15.70**	**0.03**
	Daytime	Average	75.45 ± 6.96	75.16 ± 7.65	0.87
		Variability	**13.33 ± 4.25**	**11.45 ± 2.72**	**0.04**
		Minimum	55.82 ± 7.68	54.94 ± 6.98	0.63
		Maximum	**116.76 ± 22.42**	**106.45 ± 15.99**	**0.04**
	Nighttime	Average	64.03 ± 7.17	62.62 ± 5.36	0.39
		Variability	7.65 ± 4.18	6.71 ± 2.44	0.30
		Minimum	54.77 ± 5.46	53.31 ± 5.15	0.29
		Maximum	**87.84 ± 18.84**	**79.10 ± 10.46**	**0.03**

SBP, systolic blood pressure; DBP, diastolic blood pressure; MAP, mean arterial pressure; PP, pulse pressure; HR, heart rate; values are shown as mean ± SD, statistically significant differences marked as bold (*p* < 0.05).

**Table 4:** Comparison of ambulatory BP data among study groups with apnea-to-bruxism indexes < 1 and apnea-to-bruxism indexes ≥ 1.

	Variable		Apnea-to-Bruxism < 1	Apnea-to-Bruxism ≥ 1	*p*
SBP (mmHg)	24 h mean	Average	**109.28 ± 7.16**	**113.83 ± 8.95**	**0.04**
		Variability	**12.13 ± 2.92**	**13.82 ± 2.91**	**0.04**
		Minimum	84.96 ± 7.54	84.22 ± 7.34	0.73
		Maximum	139.74 ± 12.92	147.33 ± 15.83	0.05
		Decline (%)	10.45 ± 6.39	11.22 ± 5.26	0.66
	Daytime	Average	**112.57 ± 7.97**	**117.78 ± 9.84**	**0.03**
		Variability	10.74 ± 2.36	11.98 ± 3.28	0.10
		Minimum	89.87 ± 9.11	92.44 ± 10.36	0.33
		Maximum	139.74 ± 12.92	146.61 ± 15.99	0.08
	Nighttime	Average	100.53 ± 7.54	104.59 ± 8.41	0.07
		Variability	**8.88 ± 3.24**	**11.64 ± 3.30**	**0.00**
		Minimum	86.02 ± 8.38	84.35 ± 7.25	0.47
		Maximum	**119.37 ± 10.80**	**127.76 ± 15.97**	**0.02**
DBP (mmHg)	24 h mean	Average	67.93 ± 5.81	71.11 ± 6.34	0.06
		Variability	10.94 ± 2.50	11.82 ± 2.97	0.24
		Minimum	47.20 ± 6.97	45.83 ± 5.51	0.46
		Maximum	98.22 ± 13.87	103.28 ± 18.39	0.24
		Decline (%)	15.78 ± 7.97	15.69 ± 5.16	0.97
	Daytime	Average	**70.96 ± 6.40**	**75.78 ± 7.68**	**0.01**
		Variability	9.94 ± 2.56	10.10 ± 3.68	0.84
		Minimum	51.04 ± 7.49	53.50 ± 9.44	0.28
		Maximum	98.13 ± 13.97	101.56 ± 17.25	0.41
	Nighttime	Average	59.70 ± 6.64	62.88 ± 5.89	0.09
		Variability	**7.92 ± 2.85**	**9.87 ± 3.00**	**0.02**
		Minimum	47.53 ± 6.81	46.18 ± 5.39	0.47
		Maximum	**75.98 ± 8.69**	**84.00 ± 17.54**	**0.02**
MAP (mmHg)	24 h mean	Average	**82.15 ± 5.63**	**85.72 ± 6.69**	**0.03**
		Variability	10.52 ± 2.33	11.48 ± 2.58	0.16
		Minimum	61.50 ± 6.59	61.72 ± 6.42	0.90
		Maximum	111.83 ± 13.01	117.00 ± 16.27	0.19
		Decline (%)	12.49 ± 6.48	12.49 ± 4.75	1.00
	Daytime	Average	**84.98 ± 6.29**	**89.06 ± 7.39**	**0.03**
		Variability	9.62 ± 2.21	10.02 ± 3.27	0.57
		Minimum	64.74 ± 6.89	67.83 ± 8.60	0.14
		Maximum	111.61 ± 12.94	114.39 ± 16.24	0.48
	Nighttime	Average	74.40 ± 6.09	77.82 ± 6.58	0.06
		Variability	**7.48 ± 2.82**	**9.57 ± 2.97**	**0.01**
		Minimum	62.26 ± 6.81	61.94 ± 6.35	0.87
		Maximum	**89.58 ± 9.60**	**98.00 ± 16.26**	**0.02**
PP (mmHg)	24 h mean	Average	41.41 ± 4.97	42.67 ± 4.97	0.37
		Variability	7.02 ± 1.83	7.82 ± 2.17	0.14
		Minimum	23.65 ± 2.62	24.17 ± 4.50	0.57
		Maximum	61.28 ± 9.94	63.33 ± 10.17	0.46
	Daytime	Average	41.61 ± 5.14	43.06 ± 5.40	0.32
		Variability	7.25 ± 1.98	7.95 ± 2.26	0.23
		Minimum	24.17 ± 3.84	25.22 ± 4.93	0.37
		Maximum	60.52 ± 10.08	62.22 ± 10.01	0.55
	Nighttime	Average	40.86 ± 5.04	41.47 ± 5.15	0.68
		Variability	**5.50 ± 1.52**	**6.84 ± 2.16**	**0.01**
		Minimum	29.86 ± 4.95	28.06 ± 5.51	0.22
		Maximum	**52.16 ± 7.92**	**57.29 ± 8.39**	**0.03**
HR (beats/min)	24 h mean	Average	72.74 ± 5.63	70.44 ± 8.58	0.21
		Variability	**13.39 ± 3.04**	**11.34 ± 3.48**	**0.02**
		Minimum	53.30 ± 5.70	51.78 ± 6.68	0.36
		Maximum	**116.00 ± 20.41**	**103.72 ± 18.40**	**0.03**
	Daytime	Average	75.89 ± 6.06	73.83 ± 9.69	0.31
		Variability	**13.06 ± 3.47**	**10.77 ± 3.82**	**0.02**
		Minimum	56.02 ± 6.60	53.78 ± 8.85	0.27
		Maximum	**115.24 ± 19.75**	**102.89 ± 18.67**	**0.03**
	Nighttime	Average	64.14 ± 6.16	61.35 ± 6.56	0.13
		Variability	7.67 ± 3.68	5.99 ± 2.53	0.09
		Minimum	54.44 ± 5.29	53.12 ± 5.43	0.39
		Maximum	**86.56 ± 16.42**	**76.18 ± 11.78**	**0.02**

SBP, systolic blood pressure; DBP, diastolic blood pressure; MAP, mean arterial pressure; PP, pulse pressure; HR, heart rate; values are shown as mean ± SD, statistically significant differences marked as bold (*p* < 0.05).

**Table 5:** Results of estimation for the final model obtained on multivariate regression analysis. model for average MAP (24 h Mean).

Parameter	Model for Average MAP (24 h Mean)		
	Rc	SEM of RC	*p*
Intercept	**68.31**	**4.72**	**0.000**
Apnea-to-bruxism index	**0.44**	**0.23**	**0.045**
Male gender	0.66	1.62	0.685
BMI	**0.53**	**0.26**	**0.041**
Age	0.06	0.09	0.472

MAP, mean arterial pressure (mmHg); BMI, body mass index (kg/m^2^); Rc, regression coefficient; SEM, standard error of mean; statistically significant differences are marked as bold (*p* < 0.05).

**Table 6:** Results of the estimation for the final model obtained on multivariate regression analysis. Model for average daytime MAP.

Parameter	Model for Average MAP (Daytime)		
	Rc	SEM of RC	*p*
Intercept	**69.01**	**5.14**	**0.000**
Apnea-to-bruxism index	**0.72**	**0.30**	**0.048**
Male gender	1.28	1.76	0.468
BMI	**0.59**	**0.28**	**0.036**
Age	0.08	0.10	0.424

MAP, mean arterial pressure (mmHg); BMI, body mass index (kg/m^2^); Rc, regression coefficient; SEM, standard error of mean; statistically significant differences are marked as bold (*p* < 0.05).

**Table 7:** Results of the estimation for the final model obtained on multivariate regression analysis. Model for nighttime SBP variability and anthropometric and polysomnographic characteristics.

Parameter	Model for SBP Variability (Nighttime)		
	Rc	SEM of RC	*p*
Intercept	**8.49**	**2.64**	**0.002**
Apnea-to-bruxism index	**0.33**	**0.11**	**0.040**
Male gender	**2.08**	**0.90**	**0.025**
BMI	−0.05	0.14	0.724
Age	0.04	0.05	0.434

SBP, systolic blood pressure (mmHg); BMI, body mass index (kg/m^2^); Rc, regression coefficient; SEM, standard error of mean; statistically significant differences are marked as bold (*p* < 0.05).

**Table 8:** Results of the estimation for the final model obtained on multivariate regression analysis. Model for nighttime SBP variability and anthropometric, bruxism, and polysomnographic indices.

Parameter	Model for SBP Variability (Nighttime)		
	Rc	SEM of RC	*p*
Intercept	**11.48**	**3.76**	**0.004**
BEI > 4	**1.57**	**0.63**	**0.039**
BMI ≥ 25	**2.03**	**1.00**	**0.042**
Arousals	**0.18**	**0.09**	**0.044**
TST	**−0.01**	**0.01**	**0.026**

SBP, systolic blood pressure (mmHg); BEI, bruxism episode index (*n*/hour); BMI, body mass index (kg/m^2^); TST, total sleep time; Rc, regression coefficient; SEM, standard error of mean; statistically significant differences are marked as bold (*p* < 0.05).

## Data Availability

The data presented in this study are available on request from the corresponding author. The data are not publicly available due to European Union restrictions.
